# Marine heatwaves and the collapse of marginal North Atlantic kelp forests

**DOI:** 10.1038/s41598-020-70273-x

**Published:** 2020-08-07

**Authors:** K. Filbee-Dexter, T. Wernberg, S. P. Grace, J. Thormar, S. Fredriksen, C. N. Narvaez, C. J. Feehan, K. M. Norderhaug

**Affiliations:** 1 Institute of Marine Research, Nye Flødevigveien 20, 4817 His, Norway; 2 UWA Oceans Institute and School of Biological Sciences, The University of Western Australia, Perth, Australia; 3 Department of Biology and Werth Center for Coastal and Marine Studies, Southern Connecticut State University, New Haven, CT 06515 USA; 4 Department of Biosciences, University of Oslo, Blindern, PO Box 1066, 0316 Oslo, Norway; 5 Department of Biology, Villanova University, Villanova, PA 19085 USA; 6 Department of Biology, Montclair State University, Montclair, NJ 07043 USA

**Keywords:** Climate-change ecology, Ecosystem ecology, Marine biology, Climate-change impacts

## Abstract

Extreme climatic events including marine heatwaves (MHWs) are becoming more frequent and severe in the Anthropocene. However, our understanding of how these events affect population dynamics of ecologically important species is limited, in part because extreme events are rare and difficult to predict. Here, we quantified the occurrence and severity of MHWs over 60 years in warm range edge kelp forests on both sides of the North Atlantic. The cumulative annual intensity of MHWs increased two- to four-fold during this period, coinciding with the disappearance of kelps. We experimentally demonstrated a relationship between strong and severe 2018 heatwaves and high kelp mortality in both regions. Patterns of kelp mortality were strongly linked to maximum temperature anomalies, which crossed lethal thresholds in both regions. Translocation and tagging experiments revealed similar kelp mortality rates on reefs dominated by healthy kelp forests and degraded sediment-laden algal ‘turfs’, indicating equal vulnerability to extreme events. These results suggest a mechanistic link between MHWs and broad-scale kelp loss, and highlight how warming can make ecosystem boundaries unstable, forcing shifts to undesirable ecosystem states under episodically extreme climatic conditions.

## Introduction

Extreme events may pose a stronger, more immediate threat to ecosystem function than shifts in average conditions, and are increasingly manifesting as key drivers of ecosystem reconfiguration as environmental conditions become more variable and extremes more frequent with climate change^1–3^. Discrete periods of anomalously high ocean temperatures, known as “marine heatwaves” (MHWs)^4^, can have serious consequences for ecosystems, and are often associated with loss of ecological function and services^5,6^. The increased severity and frequency of MHWs threaten biodiversity and ecosystem function on global scales^5^ and the total number of MHW days per year has increased by > 50% in recent decades^1,7,8^. Yet, the ecological consequences of extreme events such as MHWs have been identified as a key knowledge gap in ecology^9,10^, and our understanding of population dynamics during MHWs is limited^5^. This is partly because these processes are not often studied in the field because MHWs are difficult to predict, and their impacts challenging to manipulate in situ^11,12^. Compounding these problems, until recently, most ecological research on MHWs has been conducted on local scales using varying definitions of extremity, making broadscale measures and comparisons of the ecological effects of MHWs challenging^4,13^.

Temperature extremes appear to be particularly damaging for species located at their range edge, because they can abruptly push conditions beyond thermal tolerances, causing direct mortality^5,14^. Kelps are cool-water species that are broadly distributed and respond strongly to changing abiotic conditions^15^. Temperature is the most important factor controlling the range distribution of kelps^16–18^, and kelp forests in many regions have been shown to be vulnerable to warming^19–25^. When temperatures are already above the thermal optimum, warming will have direct negative effects on kelps, such as tissue damage, increased dislodgement, reduced photosynthetic performance, reduced reproduction and reduced growth^14, 26–28^. Negative effects of high temperatures are not always immediately visible, and can include stress-induced depletion of nutrient reserves and reduced metabolic capacity at the individual level, or reduced genetic diversity at the population scale, all of which can reduce performance of species in the long-term, creating lagged responses to temperature extremes^27,29,30^. Alternatively, natural selection following disturbance events can result in reduced impacts of future events on marine populations (i.e., ‘ecological memory’)^31^.

Globally, at many warm range margins of kelp forests, there has been an accelerating loss of kelp and an associated rise and persistence of degraded, sediment-laden algal ‘turfs’^32^. Such habitat shifts have serious consequences for ecosystem services^33–35^. In the North Atlantic, kelps forests extend from southern New England in the eastern USA, northward through eastern Canada, along Greenland and Svalbard, and along the western coast of Europe to the southernmost distribution in Portugal^36^. A localized range edge for kelp occurs in the Skagerrak in southern Norway, at the middle of their European distribution, because the upper layers of this water body are warmer than the surrounding North Atlantic^37^.

Kelp forests in southern Norway are dominated by *S. latissima* in moderately-exposed to sheltered regions, and by *Laminaria digitata* in more exposed regions of the coast. These forests are the dominant marine habitat in the subtidal zone, which is mainly bedrock shelves and fjords, and form extensive habitats around the southern tip of Norway and northward along the coast. Kelp forests in the eastern USA also are characterized by *S. latissima* and *L. digitata* and occur on sloping bedrock and boulder bottom along inshore coasts and at offshore granite ledges, with *S. latissima* dominating at the southernmost (warm) range limit in Rhode Island Sound and Long Island Sound. Adult *S. latissima* typically live up to 3 years and undergo a period of fast growth in spring and early summer followed by slow growth and erosion in late summer and autumn^38,39^.

Here we explore the impact of MHWs on *S. latissima* kelp forests at warm range edges on both sides of the North Atlantic. Our study areas are on the border of the Skagerrak in southern Norway, and around Rhode Island Sound and Long Island Sound in southern New England, eastern USA. Both areas were historically dominated by kelp^40–43^, and have experienced declines of kelp forests over the last two decades, and a corresponding increased dominance by turf algae^42,44^. These declines in both southern Norway and the eastern USA have been associated with warming temperatures, but the role of MHWs is unclear. The occurrence of MHWs in both regions during 2018 provided a unique opportunity to track and compare kelp mortality during these events simultaneously in two regions. Using a combination of in situ measures of temperature and kelp abundance, translocation experiments, and historical records we asked three questions: (1) Are long-term patterns of kelp forest decline associated with an increasing occurrence of MHWs?, (2) accordingly, do discrete MHW events (2018) result in kelp loss?, and (3) are kelps inhabiting degraded (turf-dominated) reefs more or less vulnerable to mortality than kelps inhabiting healthy (kelp-dominated) reefs during MHWs?

## Results

### Extreme temperatures and marine heatwaves

**Figure 1:**
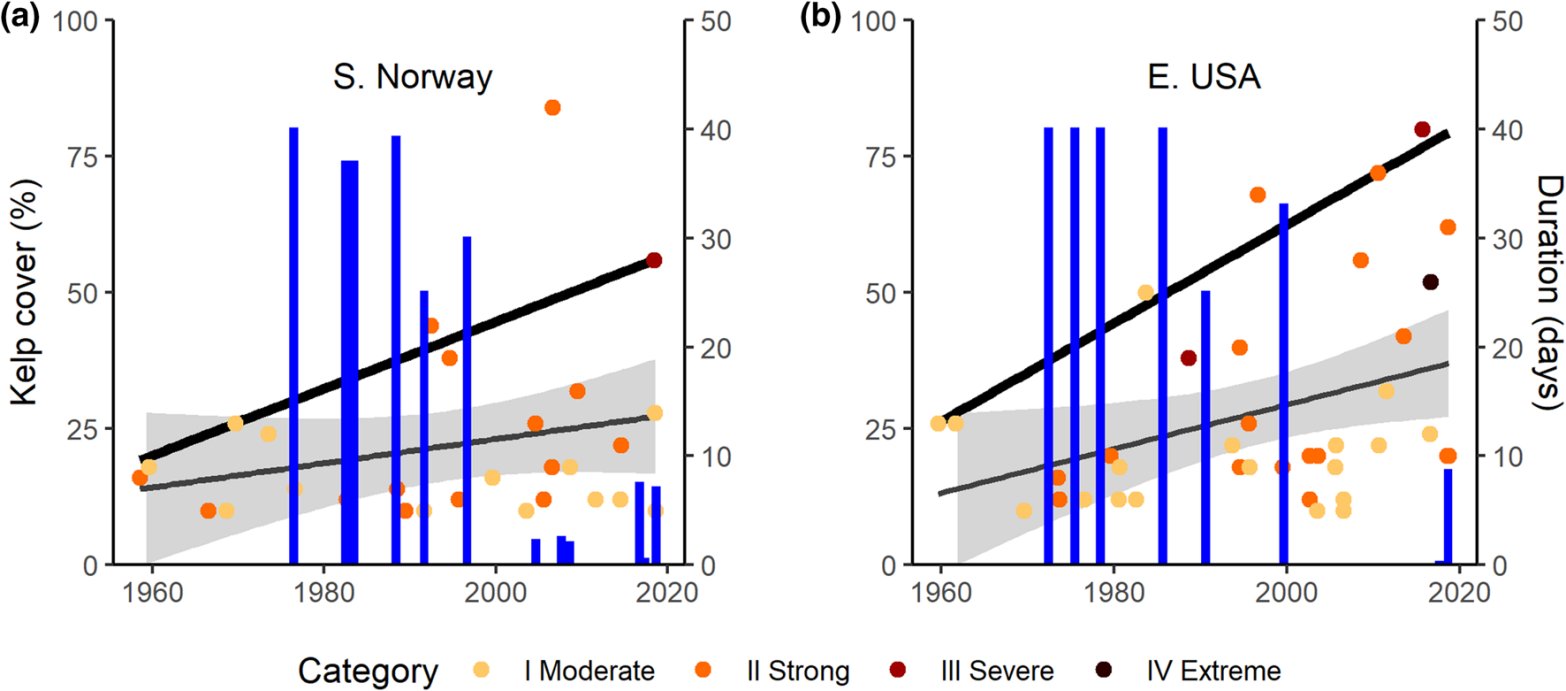
Historic kelp cover (blue bars) and occurrence of MHWs (points) that equaled or exceeded temperature thresholds for mortality of *S. latissima* in southern Norway (19.7 °C) (**a**) and the eastern USA (22.8 °C) (**b**). Colours indicate the category of MHW severity (based on size of °C anomaly relative to the climatological mean). Trendlines are fitted using a linear model (gray) with 95% confidence interval. Black line is quantile regression showing change in the 90th quantile of the predictor variable. Temperature data are from subsurface loggers. Kelp cover data and errors are reported in Supplementary Table S2.

Over the past 30 years (1989–2018) the annual cumulative intensity of MHWs (summed intensity of all heatwaves in each year from subsurface temperature records) has increased 340% in southern Norway (36.4 degree days ± 20 to 126 degree days ± 109; mean ± SD) and 228% in the eastern USA (58.3 degree days ± 32 to 132.1 degree days ± 98; mean ± SD) compared to the preceding 30 years (1959–1988) (Supplementary Fig. S1). The frequency of MHW events each year increased between 1959–2018 at a rate of 0.028 events year^−1^ in southern Norway, which is a rise from 1.4 to 3.1 MHWs year^−1^ (LM: R^2^ = 0.12, p = 0.002; Fig. 1a) and at a rate of 0.056 year^−1^ in the eastern USA, which is a rise from 0.5 to 3.9 MHWs year^−1^ (LM: R^2^ = 0.27, p < 0.001; Fig. 1b). Moreover, over this 60-year record, most severe and extreme category heatwaves (according to MHW classification in Ref.^4^) occurred in the last two decades^13^ (Supplementary Fig. S1). Maximum annual sea temperatures in southern Norway ranged between 16 and 22 °C and increased at a rate of 0.04 ± 0.011 °C year^−1^ (mean ± SD; LM; p = 0.002, R^2^ = 0.15) over the 100-year record*.* Maximum ocean temperatures in the eastern USA fluctuated between 15 and 25 °C over the 60-year record, with a rate of increase of 0.03 ± 0.009 °C year^−1^ (LM; p = 0.001, R^2^ = 0.18), and were higher compared to southern Norway.

Temperatures in both regions during MHW events regularly exceeded the local mortality thresholds (19.7 °C in Norway and 22.8 °C in the eastern USA) for *S. latissima* (Fig. 1). The average duration of MHW events that crossed mortality thresholds over the past 60 years showed an increasing but insignificant trend in Norway (0.17 ± 0.16 days year^−1^; LM: R^2^ = 0.04, p = 0.305) and an increasing trend in the eastern USA (0.20 ± 0.09 days year^−1^; R^2^ = 0.13, p = 0.029). The duration of the most anomalous MHW events (90th quantile) increased 3.7 and 4.6× faster than average, rising by 0.63 days year^−1^ in southern Norway (Fig. 1a) and 0.91 days year^−1^ in the eastern USA (Fig. 1b).

Historic records of kelp cover for both study areas showed kelp losses over the last 2 decades. Kelp cover declined from 82% ± 9 to 17% ± 9 (mean ± SD) between 1982 and 2018 in southern Norway, with the sharpest reduction of cover (− 55%) occurring between 1996 and 2004 (Fig. 1a). Kelp cover was variable between 2004 and 2018, fluctuating between 0 and 15%, but remained well below historic levels of dominance. Similarly, kelp cover declined from historic observations of full cover (~ 80%) to 4.4% ± 4.4 (mean ± SD) between 1972 and 2018 in the eastern USA, with the sharpest reduction of cover (− 65%) occurring between 1999 and 2017 (Fig. 1b). Overall, the infrequent historic sampling for these areas (particularly in eastern USA in the 2000s), make it difficult to test whether the timing of increasingly frequent and severe of MHWs was related to the onset of kelp forest loss in these areas. Yet, large drops in kelp cover on both sides of the North Atlantic coincided with years with large cumulative heatwave intensity and longer periods above temperature mortality thresholds.

**Figure 2:**
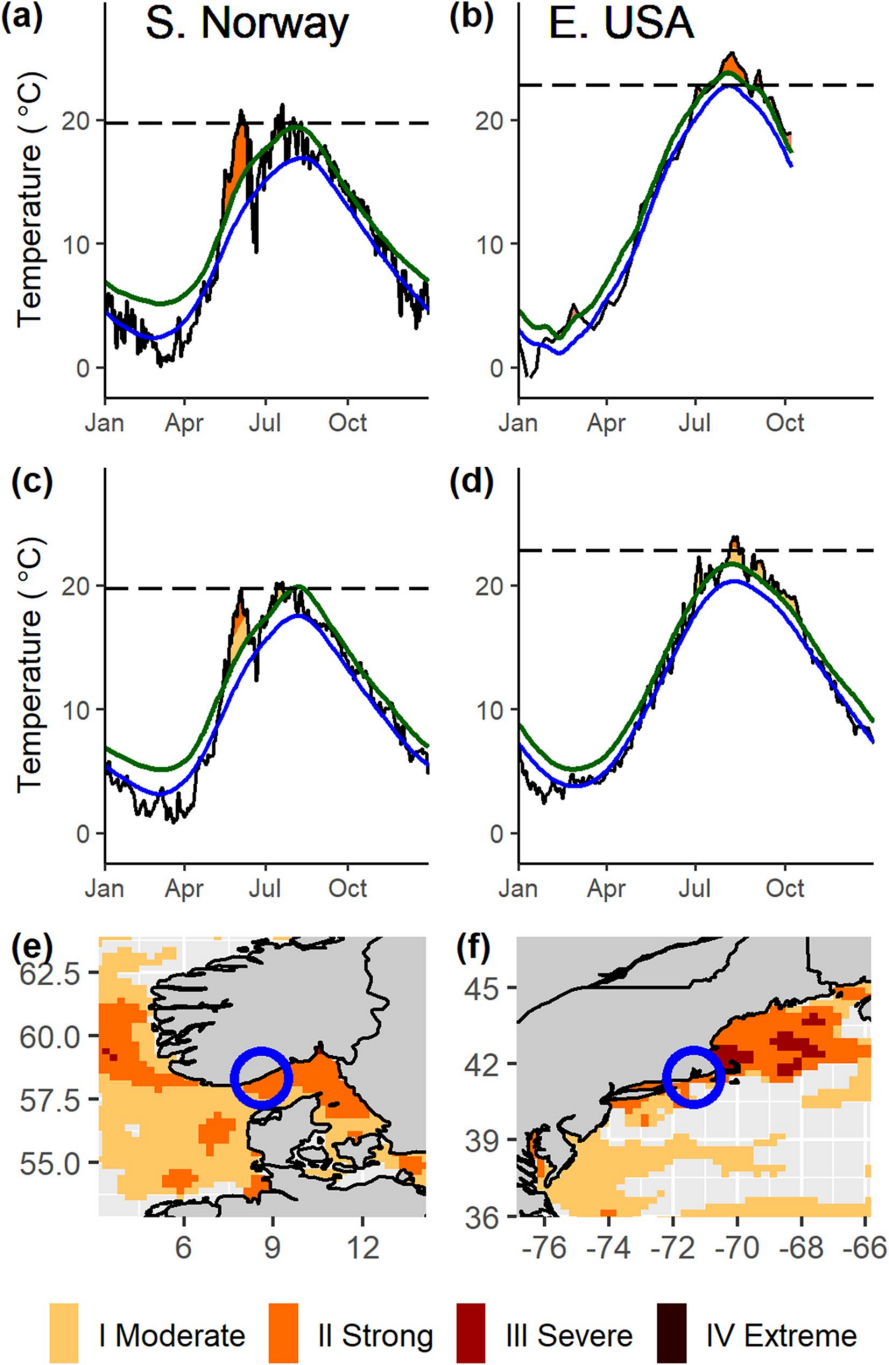
Details of MHW events based on long-term temperature records from subsurface temperature loggers (**a**,**b**) and satellite-derived SST measures (**c**,**d**). Black lines show 2018 temperatures. Green lines are the MHW threshold temperature, calculated using the 90th percentile above the 30-year climatology record (blue line). Dashed line indicates lethal temperature threshold for sugar kelp. Maps in bottom panels show spatial extent of the 2018 MHW event around S. Norway (**e**) and E. USA (**f**) study areas (blue circles) at the time of maximum temperature in (**a**,**b**). Maps were generated in R using the HeatwaveR package (R Core Team, https://www.R-project.org, R package version 0.4.4, https://CRAN.R-project.org/package=heatwaveR), and the marine heatwave tracker^68,71^. Events are categorized into the four classes of MHW severity.

We conducted field experiments during 2018—an unusually warm year—at sites distributed along 37 km of coastline, on the border of the Skagerrak in southern Norway (Fig. 2a) and over 95 km of coast in Rhode Island Sound and Long Island Sound in the eastern USA (Fig. 2b, Supplementary Table S1). Subsurface temperature loggers recorded a severe category III MHW in southern Norway in spring (Fig. 2a) and four successive strong category II MHWs in the eastern USA in 2018 (Fig. 2b). In Norway, the MHW lasted 28 days reaching temperatures up to 20.8 °C with maximum intensity on 6 June. Three moderate (category I) MHWs with a combined duration of 19 days also occurred, peaking on 20 July, 1 August and 20 October. Maximum temperatures during the first two of these events reached 21.3 and 20.2 °C, respectively, thus exceeding local temperature thresholds for sugar kelp mortality (Fig. 2a). The MHWs in the eastern USA reached their maximum intensities on 3 July, 8 August, 4 September, and 8 October, and had a combined duration of 77 days. Mean temperatures during the first three events exceeded 22.2 °C and the maximum temperature reached 25.4 °C, which far exceeded local temperature thresholds for sugar kelp mortality (Fig. 2b). The timing of the 2018 heatwaves identified using underwater temperature records closely mirrored those identified using SST data, but the temperatures from subsurface records were slightly higher compared to SST records in the eastern USA (Fig. 2c,d). SST data showed that at its peak intensity, the most severe 2018 heatwave covered over 400 km of linear coastline in southern Norway (Fig. 2e) and over 800 km of linear coastline in the eastern USA, demonstrating the large spatial scale of these events (Fig. 2f).

### Dynamics of kelp mortality

**Figure 3:**
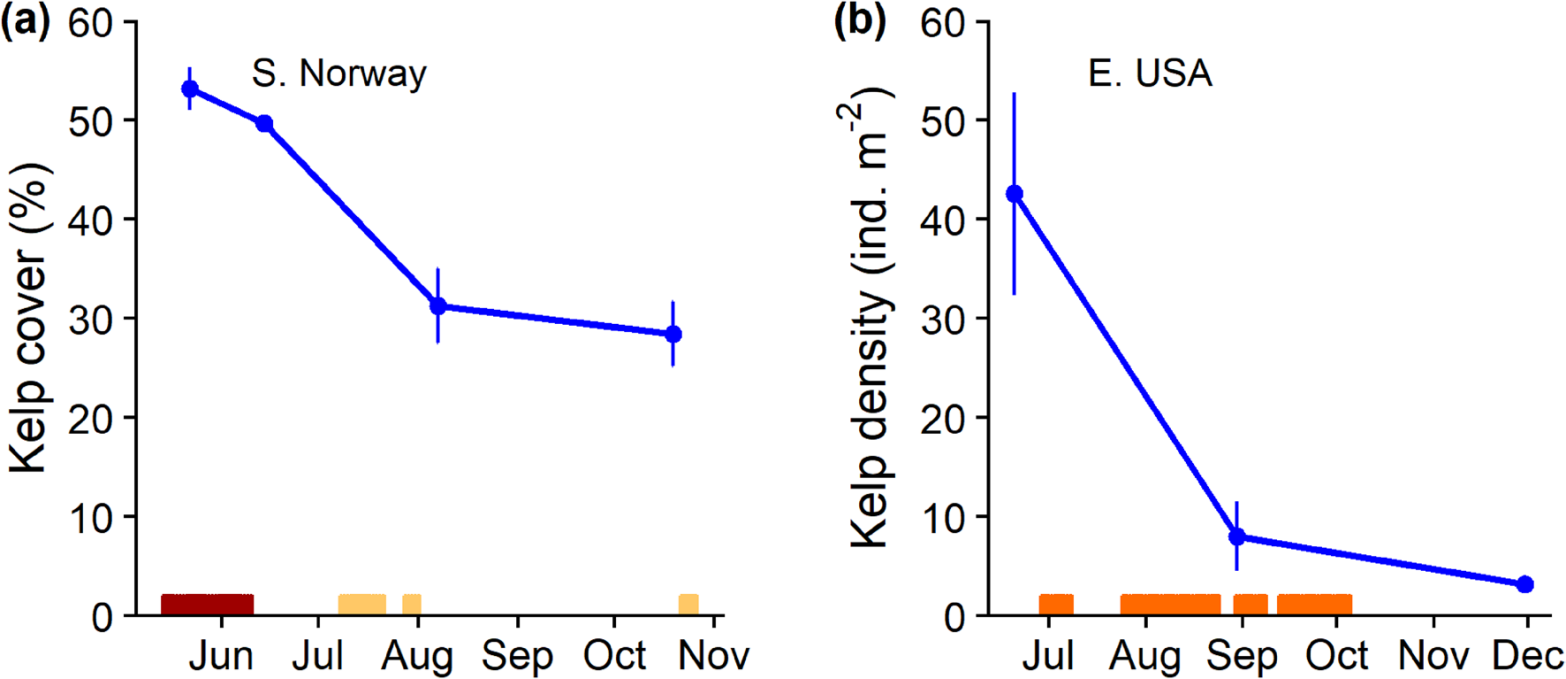
Marine heatwaves and declines in sugar kelp in southern Norway (**a**) and the eastern USA (**b**) in 2018. Horizontal bars show MHWs with colours indicating maximum severity (see Fig. 2a,b). Error bars are SDs around mean kelp cover (A) and kelp density (B). Some error bars are within the diameter of the symbol. Details on kelp sites are reported in Supplementary Table S1.

Kelp abundance was measured before, during, and immediately after the 2018 MHWs. High kelp mortality occurred at sites in both southern Norway and eastern USA following the MHWs. Kelps decreased in total blade length between August and November (77 ± 16 to 61 ± 11 cm [mean ± SD] in Norway and 33.9 ± 12.6 to 19.0 ± 11.8 cm in the eastern USA). After the MHW the cover of sugar kelp was almost halved in southern Norway, declining from 54% ± 6 before to 28% ± 10 after the event (Fig. 3a). In the eastern USA, the density of sugar kelp declined during the MHWs from 42.6 ind. m^−2^ ± 45.7 (mean ± SD) in June to 8.0 ind. m^−2^ ± 8.6 in late August, continuing to decline to 3.2 ind. m^−2^ ± 4.1 in late November (Fig. 3b). Based on population size structure in the eastern USA, kelps in June were largely recent recruits (mean ± SD blade length: 33.9 ± 12.6 cm). In Norway, average kelp growth across sites was low (0.026 cm day^−1^ ± 0.028) and average distal erosion was moderately high (0.26 cm day^−1^ ± 0.20) (± SD) during the MHW (Supplementary Table S1).

**Figure 4:**
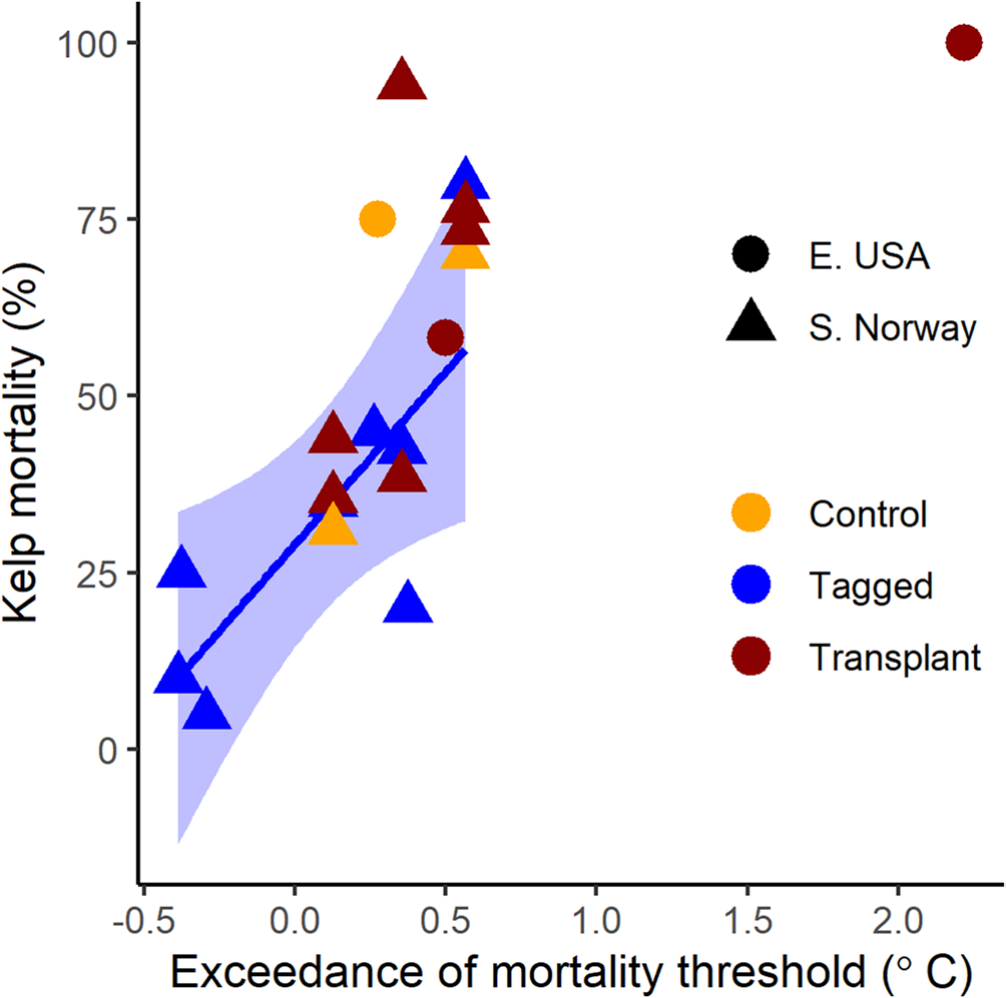
Relationship between percent mortality of transplanted and tagged kelps and the maximum sea temperatures they experienced in 2018. Controls represent kelp that were back transplanted into source sites. Temperature mortality thresholds are 19.7 °C for southern Norway and 22.8 °C for the eastern USA. Data are shown in Supplementary Table S1. Baseline mortality rates of tagged S. latissima in 1981 in S. Norway was 23 ± 6% and in 1980 in eastern USA was 47 ± 2% (SD) (calculated by extrapolating monthly mortality rates over the 1.6- and 1.8-month study periods, respectively).

The patterns of sugar kelp mortality in 2018 for kelps tagged and monitored over the warm late summer/early autumn period were strongly linked to maximum temperatures (Pearson’s R^2^ = 0.77, p = 0.025), which approached or crossed thresholds for mortality in August in both study areas (Fig. 4, Supplementary Fig. S2). At all sites where threshold temperatures were crossed by 0.5 °C or more, mortality rates exceeded 58% (Supplementary Table S1). On average, natural mortality rates of tagged kelp in southern Norway were 29.2 ± 24.9% (mean ± SD) over the 2-month tagging period (mid-August to mid-October), which is only slightly higher than average historic mortality rates of 23 ± 6% measured in 1981–82 for this species on the west coast of Norway^45^. However, the three warmest sites showed mortality rates well beyond the upper extremes of historic measures (42–80%), and mortality rates of kelp at each study site were related to how much warm temperature anomalies exceeded temperature mortality thresholds at the site (Fig. 4). In the eastern USA, the site with no remaining kelp (despite historic records of abundant kelp) and with 100% mortality of transplanted kelps exceeded temperature thresholds by over 2 °C. Light measures from loggers in Norway (not shown) were variable across sites (667 ± 293 thousand Lux; mean ± SD), but showed no relationship with kelp mortality (Pearson’s correlation coefficient = 0.46, p = 0.250). This suggests that alternative drivers such as turbidity did not affect kelp mortality and suggests that lethal temperatures were the main stressor occurring during the MHWs.

**Figure 5:**
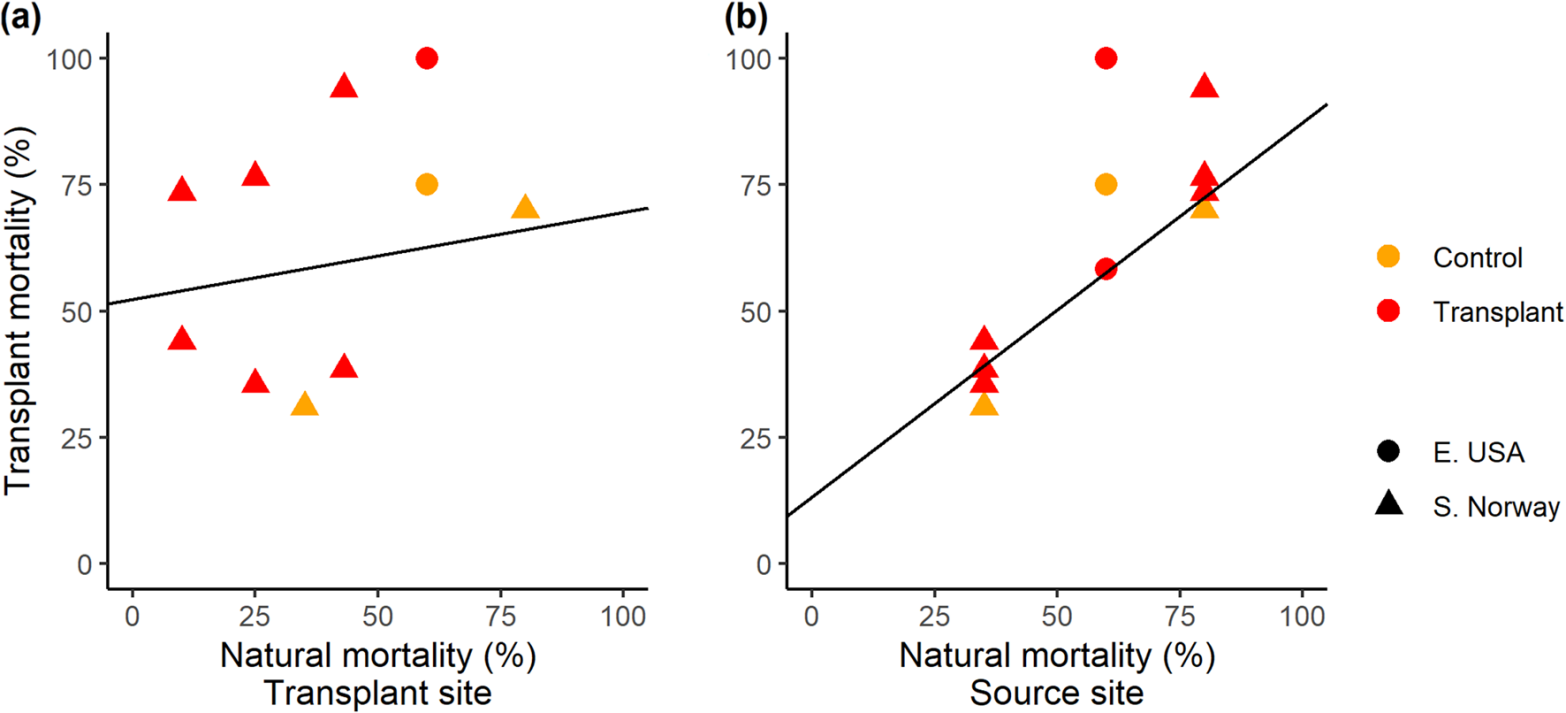
Relationships between mortality of transplanted kelps (%) and natural mortality of unmanipulated tagged kelps at the transplant site (**a**) and the source site (b). Filled circles are kelps transplanted to turf reefs (transplant) and open circles are back transplanted kelps at source sites (control). Natural mortality could not be quantified at 1 of the eastern USA transplant sites because kelp was absent. Note overlapping points at (35, 37) and (80, 75) are offset by 2%.

To test whether kelps on previously degraded reefs were equally vulnerable to extreme events compared to kelps on healthy reefs, we transplanted 10 arrays of kelps from healthy reefs to degraded reefs in both regions (1 array = 12–16 kelps secured to a 0.4 m^2^ grid). There was no support for the hypothesis that natural adult kelp mortality would be higher at degraded (turf-dominated) compared to healthy (kelp-dominated) sites (Linear Mixed Effects Model; Fixed effect = kelp cover, *t*_6_ = 0.540, p = 0.609, with random effect of Region). We recorded the highest mortality (80%) at an exposed site with high spring kelp cover of 75% (S8), and little to no mortality (0, 5 and 10%) at 3 largely turf-dominated sites (SA2, SA4, S17; Supplementary Table S1). There was also no significant relationship between the mortality rates of transplanted kelps, and natural mortality of tagged kelps in the area where we transplanted them (R^2^ = 0.18, p = 0.730) (Fig. 5a) (note these comparisons was only possible in southern Norway as natural mortality was not determined in eastern USA). This indicates that the environmental conditions or biological factors at the recipient ‘turf-dominated’ transplant sites were not the main drivers of transplant mortality. Instead, transplant mortality was strongly correlated with natural mortality rates at the source sites (R = 0.79, p = 0.003; Fig. 5b). Average mortality rates for transplanted kelps were high [60 ± 23% in southern Norway and 79 ± 21% eastern USA (mean ± SD)], yet there was no difference in mortality rates between tagged kelps and back transplanted controls at the two source sites in southern Norway (S3 = 35% natural mortality vs. 31% transplant mortality; S8 = 80% natural mortality vs. 70% transplant mortality), suggesting no effect of transplantation on mortality. A portion of the kelp transplants survived at all but the warmest eastern USA site (Fig. 4) and the growth of the transplanted kelps that survived ranged from 0 to 2.2 ± 2.9 cm on each array over the study period, and averaged 1.0 ± 1.0 cm (mean ± SD) across all arrays (Supplementary Table S1). Average blade erosion on each array ranged from 9.0 cm ± 12.4 to 25.2 cm ± 21.3 over the study period and averaged 15.2 cm ± 9.2 (SD) across all arrays (Supplementary Table S1).

Transplant mortality in Norway was not correlated with mean or maximum temperatures at the transplant sites (Pearson’s R = 0.17 and 0.15; p > 0.7). Contrary to this pattern, in the eastern USA the transplant site with 100% mortality had sustained temperatures well above the threshold for *S. latissima* mortality for 10 consecutive days, whereas temperatures at the source site and the second transplant site where kelp transplants survived were cooler (Fig. 4). These results provide further evidence that adult kelps transplanted onto turf reefs do not have reduced survival compared to adult kelps on kelp-dominated reefs.

## Discussion

Kelp forests have declined in cover, biomass and areal extent globally, and in many regions have been replaced by turf algae^19–21,44^. Often these declines and habitat transformations have been associated with increasing sea temperatures at warm range edges^21,23,44^ (but see Ref.^46,47^). However, the relative importance of gradual warming and extreme temperature events (i.e., MHWs) remain unresolved. Further, the effects of maximum temperatures often get ascribed to trends of gradual warming, but this may be an artifact of not having temporal resolution to distinguish between effects of extreme as opposed to gradual environmental change^3,48^. Here we show that declines in sugar kelp (*Saccharina latissima*) and shifts to structurally simplified turf seascapes over the last two decades in southern Norway and the eastern USA coincided with strong increases in the frequency and cumulative intensity of MHWs. Lack of annual and multi-year measures of kelp abundance prevent us from directly linking these MHWs and kelp loss, but our measures show that MHWs—driven and exacerbated by underlying warming—can push temperature conditions beyond species’ tolerance limits. Overall our results suggest that temperature conditions in both regions have fundamentally changed over the past 60–100 years, with warm periods increasing in duration and intensity, and frequently exceeding *S. latissima* mortality thresholds. These regions are both in ocean warming hotspots^49^, and are experiencing particularly strong temperature rise as anthropogenic-driven warming is coinciding with a shift in the Atlantic Multidecadal Oscillation from cool to warm phase.

Our 2018 field experiments add to growing number of regional-scale studies demonstrating how extreme warming can drive the loss of habitat-forming seaweeds^5,21,25^. Levels of mortality for tagged and transplanted kelps recorded during the 2018 MHWs were higher compared to historical measures from tagging studies from the early 1980s. In Norway, our mortality rates of 26.1% month^−1^ ± 4 SE (for all tagged and transplanted kelps, calculated by dividing average mortality rates by the 1.8 month period) were more than double the 12.5% month^−1^ ± 3 SE measured in August and September 1981^50^ and comparable to 22% month^−1^ ± 7 SE measured in August–October during warm 2006, 2007 and 2008 years^38^. It is possible that these high mortality rates were partly due to a lagged heat stress response to the spring MHW, which could have caused reduced growth and photosynthetic performance in the long-term^27^. In eastern USA, summer mortality rates of 49.1 ± 7% month^−1^ (calculated by dividing mortality rates of all tagged kelps by the 1.6 month period) are high compared to 33 ± 2 SE % month^−1^ measured in July, August, September and October in 1980 at the same sites^40^. If these mortality rates were to be repeated annually over a sustained period or occur frequently enough to prevent recovery, they would likely lead to persistent loss of sugar kelp e.g., Ref.^51^ and possibly local extinction of these habitat-forming species at these warm range edges.

We found no evidence of unusual *S. latissima* growth or erosion during the 2018 MHWs. The seasonal declines in kelp length in our study displayed typical patterns of low growth and high distal erosion reported for this species in late summer^39,45,52^. The growth rates we recorded in Norway of 0.03 cm day^−1^ ± 0.01 SD were similar to rates reported for *S. latissima* in the Skagerrak (0.04 cm day^−1^ in August and 0.01 cm day^−1^ in October 2008 at 3 m depth^38^), but slower compared to nearby measures from Denmark (0.21 cm day^−1^ ± 0.01 at 7 m depth in July and September^53^). Growth rates in the eastern USA of 0.01–0.05 cm day^−1^ were similar to historic records of 0.015 cm day^−1^ ± 0.01 SD in August^43^.

Our findings show that *S. latissima* are sensitive to small increases around their upper temperature limit and provides further support for the assertion that extreme events often drive sudden ecological change. Natural mortalities were strongly linked to maximum temperatures, which approached or exceeded known thresholds for mortality^43,54–56^. Above their thermal window of optimal performance, species respond to heat stress by acclimation and adaptation, until eventually temperatures cross a threshold where they cause cellular damage (loss of function and structure of cellular components) and acute secondary stresses (oxidation, biotic interactions)^12,26,57–59^. Importantly, the relationships between kelp mortality and exceedance of thermal thresholds were consistent between the northwestern and northeastern Atlantic, suggesting that despite differences in the mortality threshold the response to exceeding this limit appears similar across the species range.

The strong link between the mortality of transplanted kelps at the ‘turf sites’, and the mortality of unmanipulated tagged kelps and back-transplanted kelps at the ‘source sites’ where the transplants came from, suggests a lagged effect of peak temperatures following the heatwave. That is, kelp survival and growth were more closely related to the maximum temperatures they had experienced prior to being transplanted, than to the cumulative or average temperatures experienced over the experiment. This is consistent with laboratory studies showing reduced photosynthetic performance, growth, and depletion of energy stores for weeks following extreme temperature events^27,29^. In general, however, our understanding of the time scales (including potential time lags) that kelps and other plants respond to extreme environmental change is incomplete^10,58^.

Interestingly, adult kelps showed similar mortality on degraded and healthier, kelp-dominated reefs. This suggests that factors other than adult mortality are limiting the establishment and persistence of kelps on turf-dominated reefs. Research suggests that early life history processes (e.g., propagule limitation, recruitment) strongly influence recovery of kelp canopies, and these may be maintaining the turf state^56,60,61^. Alternatively, since the current levels of degradation are the result of changing environmental conditions and biological processes that have occurred over many decades, our measures in 2018 may not reflect the long-term spatial patterns of mortality. For example, sediment and nutrient loading, which are considered key secondary stressors because they cause light limitation and faster turf growth^62,63^, were both exceptionally low during 2018. In Norway, during the unusually warm 2018 spring, rapid evaporation reduced the amount of meltwater in the surrounding fjords and caused the lowest ever recorded July water level in Glomma, the main river flowing into the Skagerrak (Hege Hisdal pers comm, Norwegian Water Resources and Energy Directorate monitoring from 1902–2018). This resulted in very clear water in the Skagerrak, which could have influenced kelp performance due to increased light. In the eastern USA nutrient loading has declined substantially in the study area since 1972^44^. While this lends support to the causal relationship between temperature and kelp loss, it may have decoupled spatial patterns of mortality in 2018 with spatial patterns of past drivers of historic kelp loss due to other stressors.

Our results support previous findings that different sugar kelp mortality thresholds and temperature tolerances occur on both sides of the North Atlantic. The data from the eastern USA do not capture patterns of regional or temporal variation as well as our data from Norway. However, together, these findings provide field-based support that species are locally adapted to the temperature conditions in different areas, and that a single temperature tolerance cannot be assumed to apply over an entire species’ distribution. Field tests showing within-species variation in thermal physiology are relatively rare, and this information is essential to include in predictive models of species’ vulnerability to climate change (e.g., ecological niche or species distribution models)^64^.

Given the increasing frequency and magnitude of extreme events, understanding the escalating consequences of these events as drivers of habitat loss is a key challenge. Using a consistent MHW definition^13^ enabled us to compare and analyze multiple metrics of extremity between regions, while also making our results relevant to other systems. It enabled retrospective analyses of temperature records to identify historical extremes and understand patterns of change during these events more broadly. Yet, the most compelling result we show was the link between exceedance of local thresholds and kelp mortality. Thus, an understanding of the ecology of the system, particularly the local temperature thresholds for mortality, may be important to operationalize this MHW tool. For example, the early spring heatwave was extreme in Norway, yet did not immediately push conditions past known thresholds for mortality. Instead the moderate MHW in summer pushed temperatures past mortality thresholds and appeared to be equally if not more damaging. It is also possible that the high mortality may have been due to a lagged or cumulative effect from both events. .

We contend that the similar dynamics of kelp loss on both sides of the North Atlantic demonstrates that warm edge habitats are at risk of becoming less stable and disappearing in a world of extreme climate conditions. Given the pronounced warming trend and the significant increase in MHWs globally in recent years^7^, such impacts on valuable marine habitats are likely to become more prevalent and highlight the increasing need for rapid reporting of these events and research on their ecological consequences.

## Materials and methods

### Environmental data

We quantified the frequency and magnitude of extreme temperature events, MHWs, using long-term records of sea temperature and the MHW framework developed by Hobday et al.^4^. In this framework, MHWs are defined as periods longer than 5 days with temperatures exceeding the 90th percentile of long-term historical records (calculated using a 30-year baseline period from 1 January 1971 to 31 December 1999). MHWs were classified in four categories of severity (I–IV)^13^, based on how much temperatures exceeded baseline conditions relative to the temperature difference between the climatological mean and the climatological 90th percentile (I = 0–1, II = 1–2, III = 2–3, and IV = 3–4 times over baseline conditions). We constrained our analysis to exclude the winter period between 1 January to 1 April because we were interested in the warmest period of the year, when sea temperatures approach upper thermal tolerance limits of adult and early life stages of *S. latissima* in the northeast Atlantic (20, 20, 19 °C)^54,56,65^ and the northwest Atlantic (20, 25, 23 °C)^43,55,66^. Using average temperatures from the thermal tolerance limits reported above (S. Norway = 19.7 °C and E. USA = 22.8 °C), we calculated how frequently MHWs exceeded thermal tolerance limits of kelp over the last 60 years in each region. We obtained sub-surface sea temperature data from long-term records that spanned > 60 years (1918–2018, Flødevigen Research Station, Norway, daily measures from 1-m depth, www.imr.no/forskning/forskningsdata/temperatur_flodevigen/draw.map?boey=1; and 1959 to 2018, weekly measures from 5-m depth in Narragansett Bay, USA, Narragansett Bay Plankton Time Series, NABATS.org and web.uri.edu/plankton/). We compared these records to interpolated daily satellite-derived sea surface temperature (SST) records from 1981–2018, averaged over a 1° latitude by 1° longitude grid cell around each study area (NOAA OISST data; www.ncei.noaa.gov/erddap/). This comparison enabled us to examine the spatial extent of the 2018 MHWs using SST records for the broader coastal area.

During field experiments in 2018, water temperature and light (Lux) were recorded hourly by HOBO data loggers (Pendant Temp-Light or TidbiT, Onset Computer Corporation) attached to the sea floor. Loggers were placed in small cleared areas (to prevent shading by the kelp canopy) and only light records for the first 2 weeks of deployment were used to ensure fouling did not confound measurements over time. Loggers were deployed at 6–7 m depth at nine sites in southern Norway (7–26 August to 5–7 November) and at 3 m depth at two sites in the eastern USA (9–14 September to 26 October). This was within the depth range of kelp forests in both regions.

### Kelp abundance

Historical records of kelp percent cover were obtained from published and gray literature and anecdotal reports for both study areas (Supplementary Table S2) and compared to temporal patterns of MHW intensity. Kelp percent cover was measured at nine sites in Norway before (April), during (July, August), and after (October, November) the 2018 MHW using drop cameras. In the eastern USA, kelp density was measured at one site with kelp present (Supplementary Table S1) before (June), during (August) and after (November) the 2018 MHW. Density rather than percent cover was used to examine changes in kelp abundance in the eastern USA, because these data were initially collected as part of different monitoring programs in each region^44^, and because density captured the high number of recruits in June in the eastern USA better than cover. However, kelp cover was measured in the eastern USA in December 2017 and February, March, and June 2018 to extend the historical record (Supplementary Table S2). Kelp cover was measured either with drop cameras from a research vessel (Norway) or by divers with an underwater camera (USA). In Norway, we conducted two perpendicular video transects at each site, one beginning at the lower depth limit of kelp and extending to the intertidal and one extending for ~ 25 m along the 5 m depth contour (range = 4 and 9 m depth). In the eastern USA, we ran a 30 m video transect at 2–6 m depth with a GoPro Hero 4 camera positioned ~ 0.5 m above the sea floor. To estimate the percent cover of kelp from videos in both regions, photo quadrats of reef were extracted (n = 10–12 per site) and analyzed with the point-count method. In cases where poor resolution rendered an image or a section of an image unreadable, the image was discarded, and another used. In the eastern USA, kelp density was measured by divers within twenty 0.25 m^2^ quadrats placed randomly along two 30-m long transect lines at 2–6 m depth.

To quantify kelp growth, erosion and mortality during peak temperatures, divers tagged 20 adult kelps at each of the 9 sites in Norway in August 2018. Individual kelps were tagged with a uniquely numbered cable-tie around their stipe and blade lengths measured. Divers punched 2 holes at the base of the blade to measure growth (5 and 10 cm above the meristem). Sites were revisited in October 2018 and divers located tagged kelps using “mud maps” of their locations relative to a fixed subsurface float (used to mark the site and attach the temperature logger). The area was searched for a minimum of 30 min to ensure no tags were missed during resampling. For all recovered kelp, divers measured the blade length and the distance from the meristem to each of the punched holes. Growth was calculated as the increase in distance from the 5 cm hole to the meristem between sampling times (for tattered blades the 10 cm hole was used)^67^. Erosion was calculated as the difference between the blade length between the two sampling times, accounting for any growth at the basal end. Mortality was recorded as the number of tagged kelps lost plus the number of tagged stipes with no blades. Kelps in the eastern USA were not tagged to measure natural mortality. Kelp lengths were recorded by divers in the eastern USA in the same quadrats as the kelp densities in June, August and November to examine population size structure during peak temperatures.

### Experimentally transplanted kelps

To determine if kelp survival differed in turf-dominated compared to kelp-dominated areas, we measured survival rates of adults transplanted from healthy reefs to turf-dominated reefs. We transplanted 10 arrays of adult kelps from sites with moderate to high kelp cover (> 33%) to ‘turf’ sites with low kelp cover (< 5%) or sites with 0% kelp cover that were historically dominated by kelp. Each transplant array consisted of 12–16 adult kelps collected haphazardly from the area surrounding the subsurface temperature logger and attached to a 0.5 m by 0.8 m metal grid by their haptera (using 3–4 small cable ties per kelp). We conducted these experiments from the 22 August to 15 October using two source ‘kelp’ sites and 3 recipient ‘turf’ sites in Norway and from 7 September to 26 October using one source ‘kelp’ site and 2 recipient ‘turf’ sites in the eastern USA (Supplementary Table S1). To allow for comparable assessment across regions, the experiment in the USA was deployed at our earliest opportunity (early September) following deployment of the experiment in Norway (late August). Fewer sites were sampled in the eastern USA than southern Norway due to logistical constraints and limited shore access. For each of the three source sites, one additional array was back-transplanted after being brought to the surface for 1–2 h as a control for mortality due to transport and transplantation. Each kelp was tagged with a numbered cable tie, measured and punched (using the method described above for in situ kelps) to measure mortality, growth and erosion following transplantation.

All statistical analyses were performed in R 3.5.1. MHW classifications, figures and maps were created using the ‘heatwaveR’ package^68^. We used linear models and quantile regression models (90%) to show temporal trends in the average duration and maximum duration of MHWs in southern Norway and the eastern USA; e.g.,^69^. We used generalized additive models to show temporal trends in the cumulative heatwave intensity (i.e., the size of the temperature anomaly in °C days) over the same period, because these measures were nonlinear. We elected not to model temporal trends in MHWs against historic records of kelp cover, because our data had long periods without records, and were therefore inappropriate to use in a time-series analysis. For the 2018 experiments in Norway, we used Pearson’s correlation tests to explore relationships between maximum temperatures, light at the seafloor, and natural mortality (% tagged kelps found dead at each site). We also used Pearson’s correlation tests to detect relationships between the mortality rates of transplanted kelps and natural mortality of tagged kelps in areas where we transplanted them, and at the source sites where the transplants came from. A linear mixed effects model was used to test for differences in kelp mortality at degraded compared to healthy sites, with a random effect of region (southern Norway; eastern USA) and a fixed effect of reef state (turf-dominated; kelp-dominated). The model was fitted using the package lme4^70^. Model assumptions were verified by plotting residuals against fitted values and against covariates in the model.

## Supplementary information

Supplementary Information.
