# Supplementary material for: Marine heatwaves and the collapse of marginal North Atlantic kelp forests
Source: Sci Rep. 2020 Aug 7;10:13388. doi: 10.1038/s41598-020-70273-x (PMC7414212; doi:10.1038/s41598-020-70273-x)
Supplement: Supplementary file 1 — Supplementary Information. [file 41598_2020_70273_MOESM1_ESM.pdf]

Electronic supplementary material

Marine heatwaves and the collapse of marginal North Atlantic kelp forests

Filbee-Dexter K, Wernberg T, Grace SP, Thormar J, Fredriksen S, Narvaez CN, Feehan CJ, Norderhaug KM

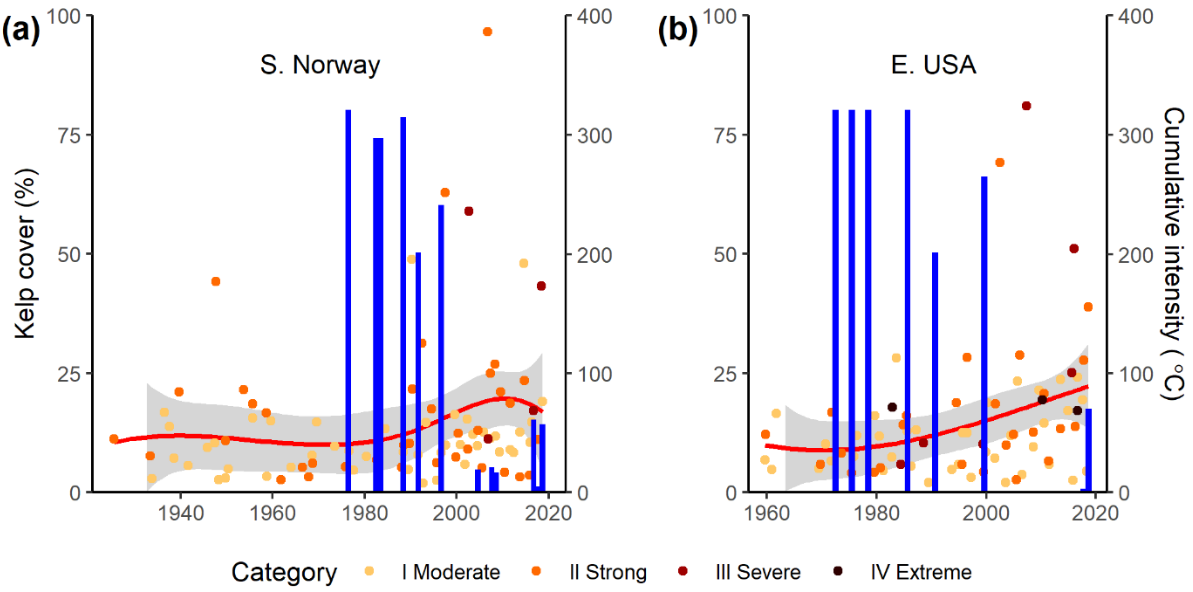

Figure S1. Historic kelp cover (blue bars) and cumulative annual intensity (°C days) of MHWs (points) for southern Norway (a) and the eastern USA (b). Colours indicate strongest category of MHW intensity each year (based on size of °C anomaly relative to the climatological mean). The cumulative heatwave intensity in each year (secondary axis) is the integral of the °C increase over baseline conditions for the duration of the events. Trendlines are fitted using a generalized additive model with thin plate regression spline smoothers (formula: y ~ s(x, 4)). Temperature data are from subsurface loggers. Of the 152 individual MHW events detected in the temperature record from southern Norway, the only severe (category III) MHWs occurred in 2002, 2016, and 2018. In the eastern USA, of the 169 individual MHW events detected over the last 60 years (1959 – 2018), severe MHWs occurred in 1984, 1988, 1999, 2007, 2015, and 2016, and extreme (category IV) MHWs in 1982, 2010, and 2016. Kelp cover data and errors are reported in Table S2.

Table S1. Average kelp cover (August in Norway and June in E USA), mortality, growth and erosion rates at study sites during 2018 MHWs. Transplant mortality is for arrays at recipient site with source site noted in brackets. Natural mortality is for tagged, unmanipulated plants at Norway sites. ** denotes kelps from sites that exceeded mortality thresholds by 0.5 °C.

Site name	Transplant type	Position latitude, longitude	Kelp cover % (SD)	Transplant mortality % [source site]	Natural mortality %	Growth mm d^{-1} (SD)	Erosion mm d^{-1} (SD)	Temp-erature logger
**S. Norway**								
SA2		58.279 ºN, 8.532 ºE	5.3 (11.3)	-	5	0.17 (0.21)	3.20 (0.36)	subsurface float
SA4	Recipient	58,312 ºN, 8.575 ºE	4.6 (5.9)	44 [S3]<br>75 [S8]**	10	0.96 (2.33)	1.31 (0.36)	subsurface float
S3	Source	58.397 ºN, 8.743 ºE	55.2 (38.0)	31 [S3]	35	0.38 (0.26)	0.63 (0.26)	subsurface float
S17	Recipient	58.421 ºN, 8.772 ºE	19.2 (20.4)	-	0	0.22 (0.18)	5.01 (0.47)	lost
S7		58.521 ºN, 8.931 ºE	21.3 (18.9)	-	45	0.01 (0.00)	2.75 (0.41)	subsurface float
S8	Source	58.512 ºN, 8.937 ºE	36.8 (30.8)	70 [S8]**	80**	0.05 (0.07)	0.88 (0.26)	subsurface float
S5	Recipient	58.505 ºN, 8.895 ºE	0.5 (2.3)	38 [S3]<br>94 [S8]**	42.5	0.22 (0.06)	5.71 (0.14)	subsurface float
S13		58.423 ºN, 8.757 ºE	19.2 (20.4)	37 [S3]<br>76 [S8]**	25	0.20 (0.01)	3.34 (0.34)	subsurface float
HB7		58.254 ºN, 8.523 ºE	73.7 (25.9)	-	20	0.96 (0.22)	1.31 (0.36)	subsurface float
**E. USA**								
King's Beach	Recipient	41.453 ºN, 71.343 ºW	0[3]	58 [FW]**	no kelp	0.48 (0.48)[1]	4.90 (3.33)[1]	estimated by SST[2]
Fort Wetherill (FW)	Source	41.478 ºN, 71.362 ºW	34.5 (35.2)	75 [FW]	ND	0.10 (0.00)[1]	3.81 (1.02)[1]	anchored to seafloor
Old Kelsey Point	Recipient	41.273 ºN, 72.424 ºW	0[3]	100 [FW]**	no kelp	0	NA	anchored to seafloor

[1]Measured on transplanted kelps. [2]Estimated using temperature data from the nearby FW logger, adjusted by +0.2 ºC, which is the difference between average SST records between sites. [3]Based on no kelp observed at the site during transplantation experiment. ND = no data. NA = not applicable

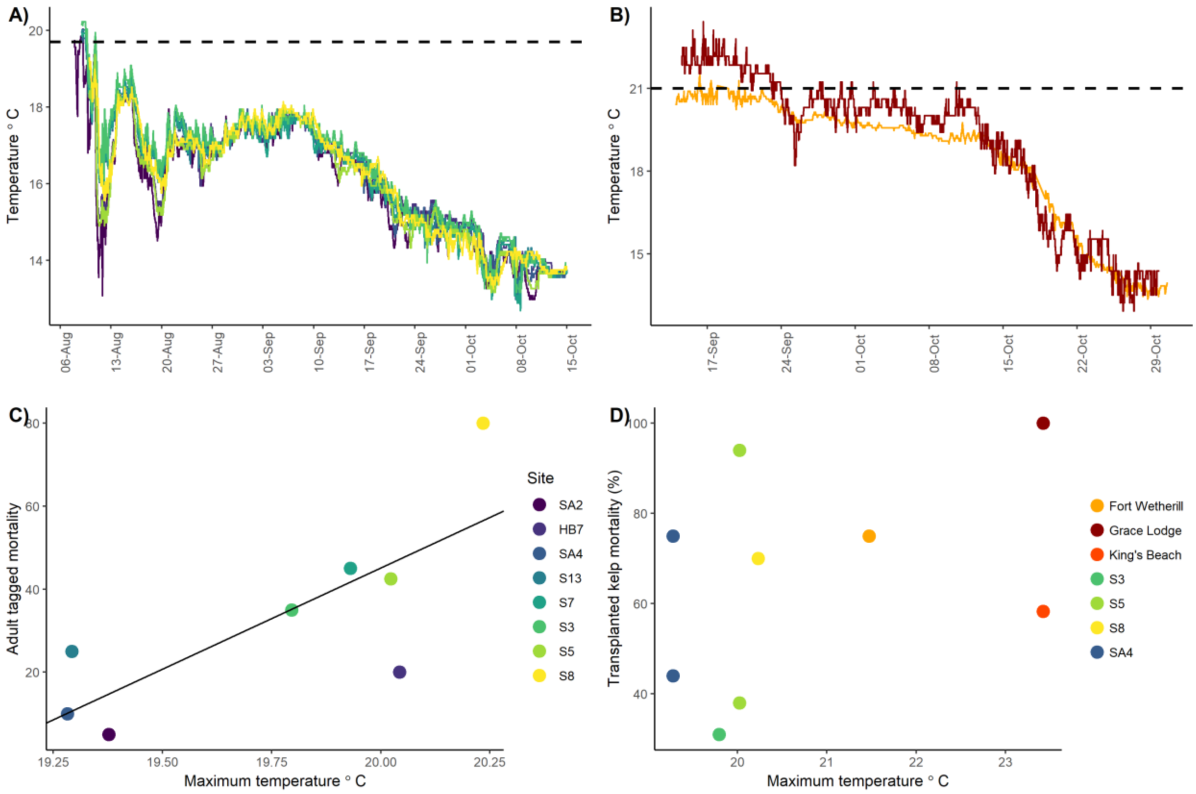

Figure S2. Water temperature measured from subsurface loggers over the duration of the experiments at all study sites in southern Norway (A) and the eastern USA (B). Threshold temperatures for *Saccharina latissima* mortality in each region are shown by dashed lines. Percent mortality in relation to maximum temperatures of tagged kelps at sites in Norway (C) and for all kelps on transplant arrays (including controls) in southern Norway and the eastern USA (D). Additional details are in Table S1.

Table S2. Historic records of kelp abundance for study areas in southern Norway and eastern USA. Site IDs are from original study (with corresponding site ID for this study in brackets). Anectodal accounts of kelp forests in Narragansett Bay are included to establish historic presence of kelp.

Site	Year	Site IDs (this study)	Kelp cover (%)	SD	Source	GPS (Lat, Lon)
Southern Norway						
Kristiansand	1976	5-03 (SA2)[1]	80	ND[2]	(Åsen 2006)	58.148, 8.041
Kristiansand	1982	5-03, 5-04 (SA2)[1]	82.5	10.6	(Åsen 2006)	58.115, 8.065
Kristiansand	1983	5-03, 5-04 (SA2)[1]	82.5	10.6	(Åsen 2006)	58.148, 8.041
Ærøy	1988	4B	78.4	3.9	(Berge et al. 1988)	58.416, 8.779
Eydehavn	1991	4-22 (S6, S7)	50		(Pedersen et al. 1993)	58.511, 8.909
Flødevigen	1996	4-8 (S13)	60	ND	(Christie 1997)	58.729, 9.287
Flødevigen/-Eydehavn	2004	4-22, 4-08 (S13, S6, S7)	4.5	0.71	(Moy and Christie 2012)	58.510, 8.909
Eydehavn	2007	4-22, (S6, S7)	5	0	(Moy and Christie 2012)	58.510, 8.909
Flødevigen	2008	4-08 (S13)	4	0	(Moy and Christie 2012)	58.729, 9.287
Grimstad	2016	SA2, SA4	15.0	14.0	(Torp 2018)	58.279, 8.532
Grimstad	2017	SA2, SA4	0	0	(Torp 2018)	58.279, 8.532
Flødevigen/ Eydehavn/Grimstad	2018	S7, S13, SA2, SA4	12.6	8.8	This study	58.728, 09.287
Eastern USA						
Narragansett Bay	1972	FW	~80	ND[3]	Wes Pratt, pers. comm.	41.478, -71.362
Narragansett Bay	1975	FW	~80	ND[3]	Wes Pratt, pers. comm.	41.478, -71.362
Narragansett Bay	1978	FW	~80	ND[3]	Wes Pratt, pers. comm.	41.478, -71.362
Narragansett Bay	1985	FW	~80	ND[3]	Sean Grace pers. obs.	41.478, -71.362
Narragansett Bay	1990	FW	>50	ND[2]	(Harlin and Rines 1993)	41.478, -71.362
Narragansett Bay	1999	FW	66	12	(Grace 2004)	41.478, -71.362
Narragansett Bay	2017	FW	0.5	1.6	This study	41.478, -71.362
Narragansett Bay	2018	FW	17.3	15.1	This study	41.478, -71.362

[1]20 km W of SA2. [2]Errors not reported in study. [3]Personal observations of researchers from the area.
